# Human-like, Animal-like, or Object-like? The Impact of LLM-Based Virtual Doctor Avatar Design on User Emotion, Physiology, and Experience

**DOI:** 10.3390/bs16030349

**Published:** 2026-03-01

**Authors:** Han Zhang, Shiyi Wang, Rui Peng

**Affiliations:** Intelligent Design, Platform at the Intersection of Art and Technology, Central China Normal University, Wuhan 430079, China; sequins@mails.ccnu.edu.cn (S.W.); pengrui@mails.ccnu.edu.cn (R.P.)

**Keywords:** virtual doctor avatars, large language models, user experience, emotional responses, social presence, digital mental health

## Abstract

Virtual agents powered by large language models are increasingly deployed in digital mental health services, yet the influence of avatar appearance on users’ emotional, cognitive, and physiological responses remains insufficiently understood. This study was conducted between March and April 2024 and examined how three avatar designs—animal-like, human-like, and object-like—shape affective experience, user evaluation, autonomic activity, and attentional allocation during virtual doctor interactions. Forty-two participants completed a within-subjects experiment involving self-reported affect ratings, multidimensional user-experience assessments, heart rate variability (HRV) measures, and eye-tracking indicators. The avatar type did not yield statistically significant differences in changes in positive or negative affect across conditions. However, physiological data revealed clear divergences. The animal-like avatar elicited the strongest parasympathetic activation, reflected by significant increases in the root mean square of successive differences (RMSSD) and high-frequency (HF) power, whereas the object-like avatar produced a sympathetic-dominant response. Across six user-experience dimensions, the animal-like avatar consistently received the highest evaluations. Eye-tracking results showed faster first fixation and a longer face-directed fixation duration for the animal-like avatar, indicating stronger social attention. The human-like avatar demonstrated slightly delayed initial fixation, consistent with subtle yet nonsignificant uncanny-valley tendencies. These findings underscore the critical role of avatar visual design in shaping emotional safety, engagement, and social processing in virtual mental-health interactions.

## 1. Introduction

### 1.1. Background and Motivation

The rapid evolution of large language models (LLMs), including GPT-4, Gemini, and Llama, has significantly advanced the capabilities of conversational artificial intelligence and accelerated its integration into mental-health services (Lawrence et al., 2024; Guo et al., 2024). Beyond the functional, task-oriented chatbots of earlier decades, contemporary LLM-based agents are designed to sustain naturalistic dialogue, provide emotional support, and facilitate long-term relational communication (Haensch, 2025; Sakhrani et al., 2021). These developments have heightened the relevance of virtual conversational agents in psychological interventions, where access barriers, stigma, and a global shortage of mental-health professionals continue to limit service availability (Mohammed, 2023). Consequently, LLM-based virtual doctors are increasingly deployed to provide low-threshold, empathetic, and personalized support, offering an alternative pathway to timely emotional assistance (Cho et al., 2023; Wang et al., 2024). Given the rapid clinical uptake of such systems, understanding how design features shape user responses has become a central scientific and practical concern (Haensch, 2025).

Among the many design variables that shape human–AI interactions, the visual embodiment of a virtual agent—including its appearance, degree of anthropomorphism, and expressive cues—plays a critical role in users’ emotional experiences, trust formation, and sense of interpersonal safety (Thaler et al., 2021; Lim et al., 2024). According to the Computers as Social Actors (CASA) framework, users naturally apply social norms and interpersonal expectations to conversational agents, even when they are aware that the agent is artificial (Riek, 2015). In therapeutic contexts, such socio-cognitive responses are particularly meaningful because visual cues can facilitate (Lim et al., 2024) or hinder the establishment of emotional safety and perceived therapeutic alliance (Bilalpur et al., 2024).

Despite the growing adoption of LLM-based virtual doctors, there is still limited empirical consensus on how different forms of avatar embodiment shape users’ emotional, physiological, and attentional responses in mental-health interactions. In particular, systematic comparisons among human-like, animal-like, and object-like virtual doctors within a unified experimental framework remain scarce.

Addressing this gap is critical for developing evidence-based design principles for emotionally supportive and trustworthy virtual clinicians. The present study aims to address this need by examining the impact of avatar embodiment on user experience using a multimodal approach.

### 1.2. Related Work

Different forms of avatar embodiment elicit distinct psychological reactions. Human-like avatars may enhance social presence, but their effectiveness depends on the naturalness of their facial features and expressions (Thaler et al., 2021). When human-like avatars approach a high degree of realism but fail to fully match natural human facial qualities, users may experience discomfort associated with the uncanny valley, a phenomenon shown to reduce trust, suppress social gaze, and impair rapport (De Borst & De Gelder, 2015; Cheetham, 2011; Fraser et al., 2025). A comprehensive empirical review further synthesized evidence that perceptual mismatch and near-human realism reliably increase eeriness and are associated with delayed or reduced facial engagement in virtual characters (Kätsyri et al., 2015). These findings suggest that near-human virtual doctors may simultaneously enhance social presence while increasing evaluative tension if perceptual coherence is imperfect.

In contrast, animal-like avatars are often perceived as warm, safe, and emotionally approachable (Lim et al., 2024). Research grounded in evolutionary psychology and human–animal interaction indicates that animal representations can attenuate defensive responses, reduce anxiety, and promote emotional openness (Artemiou et al., 2021). In digital contexts, such zoomorphic agents have been associated with greater comfort and reduced interpersonal threat, potentially facilitating self-disclosure in emotionally sensitive interactions (Ichikawa et al., 2023; Sestino & D’Angelo, 2023; A. K. E. Schneider & Bubeck, 2025). Conversely, object-like avatars lack salient social cues such as faces and eyes, which may limit emotional engagement and reduce users’ willingness to relate to the agent as a social partner (Mazerant et al., 2025). Such designs may shift interaction from a socially grounded exchange to a more instrumental task-oriented engagement.

While these theoretical distinctions are well established, empirical evidence has largely examined selected embodiment contrasts rather than systematically comparing all three types within a unified healthcare setting. As summarized in Table 1, studies published within the past decade (2015–2025) have investigated avatar realism gradients, zoomorphic warmth effects, and minimal-agent designs across healthcare, social robotics, and human–AI interaction contexts. However, these investigations predominantly focus on single categories or pairwise contrasts (e.g., human-like vs. animal-like; realistic vs. abstract), leaving direct three-way comparisons relatively underexplored.

Beyond subjective evaluations, recent research highlights the importance of multimodal measurement approaches. Visual attention, indexed through eye-tracking, provides a behavioral window into social interest and affective engagement (Cheng et al., 2023; Takenaka et al., 2024). Related work in health communication and human–computer interaction (HCI) has shown that variations in avatar stylization and realism can influence trust, social presence, and comfort, particularly when contrasting more anthropomorphic designs with simplified or abstract avatars (Canales et al., 2024; Kaiser et al., 2025). However, these studies typically examine only one or two embodiment categories at a time and do not jointly compare human-like, animal-like, and object-like virtual doctors within the same mental-health consultation context, nor do they integrate subjective, physiological, and attentional measures in a unified experimental framework.

For self-reported impressions and global evaluations, visual attention provides a behavioral window into these psychological processes (Cheng et al., 2023; Capozzi & Kingstone, 2024). Eye-tracking research demonstrates that users’ facial fixations reflect social interest, emotional involvement, and trust (Bhattacharya & Gwizdka, 2018; Tsang, 2018; Kohout et al., 2023). Reduced or delayed attention toward an agent’s face can reflect discomfort, threat appraisal, or delayed initial engagement with socially salient regions (Lim et al., 2024). These processes are particularly salient when interacting with near-human avatars (Thaler et al., 2021). However, research has yet to establish how different avatar embodiments systematically shape gaze patterns in therapeutic human–AI interactions.

Physiological responses offer another critical dimension. Heart rate variability (HRV) is a well-established index of autonomic nervous system regulation and emotional arousal (Mccraty & Shaffer, 2015; Gullett et al., 2023). Higher HRV, particularly elevated root mean square of successive differences (RMSSDs) and high-frequency (HF) power, reflects parasympathetic activation associated with calmness and emotional safety, whereas lower HRV indicates heightened stress or vigilance (Shaffer & Ginsberg, 2017; Kim et al., 2018; Stephenson et al., 2021). Studies in HCI and social robotics have shown that avatar appearance can modulate physiological responses: designs perceived as threatening or uncanny increase sympathetic activation, whereas warm and friendly designs support relaxation and stress reduction (Llanes-Jurado et al., 2024). However, the physiological effects of different virtual-doctor embodiments in LLM-mediated mental-health consultations remain underexplored.

Taken together, prior work suggests that avatar embodiment influences emotional perception, visual attention, and physiological regulation. However, existing studies are often limited to single avatar categories or pairwise comparisons and rarely integrate subjective, attentional, and physiological measures within a single experimental paradigm.

Synthesizing behavioral, attentional, and physiological evidence reveals three major gaps. First, although prior studies have compared selected embodiment types (e.g., human vs. animal, or stylized human vs. abstract), direct three-way comparisons among human-like, animal-like, and object-like virtual doctor embodiments in health-oriented dialogue settings remain scarce. As a result, it remains unclear whether these theoretically grounded differences emerge consistently when multiple avatar types are evaluated side by side within the same interactional context. Second, existing research seldom integrates multimodal indicators such as self-reported affect, PANAS scores, user-experience ratings, HRV, and gaze behavior within a single experimental paradigm. Third, the mechanisms linking visual embodiment to emotional and physiological responses, particularly the explanatory role of social attention, remain insufficiently understood in LLM-based mental-health interactions.

To address these gaps, the present study examines how three avatar types influence users’ emotional experience, physiological states, and visual attention during interactions with an LLM-based virtual doctor. Specifically, we ask the following research questions:

RQ1: Does avatar design affect users’ emotional experience and overall evaluation of the virtual doctor?

RQ2: Does avatar design evoke distinct physiological response patterns, particularly in heart rate variability (HRV) indicators associated with emotional safety?

RQ3: Does avatar design induce different visual-attention patterns, and are these patterns associated with emotional or physiological differences during interaction?

Drawing on prior theoretical frameworks, we propose the following hypotheses.

**H1.**  *Avatar design significantly shapes the emotional experience and subjective evaluation, such that differences previously suggested in isolated or pairwise studies can be examined under direct comparison, with animal-like avatars eliciting the most positive emotional responses and user ratings, followed by human-like avatars, and object-like avatars receiving the lowest evaluations.*

**H2.**  *Avatar design modulates physiological responses, such that animal-like avatars elicit greater parasympathetic activation (higher RMSSD, SDNN, and HF) compared with human-like or object-like avatars, while object-like avatars elicit higher LF/HF ratios indicative of elevated stress.*

**H3.**  *Avatar design affects visual-attention patterns: animal-like avatars will elicit more facial fixations, longer fixation durations, and shorter first-fixation latencies; human-like avatars may exhibit delayed initial engagement with the facial region, and object-like avatars will receive the lowest overall gaze allocation to facial regions.*

By integrating subjective, physiological, and attentional measures within a unified experimental framework, this study advances the understanding of how visual embodiment shapes user experiences with virtual clinicians and provides evidence-based guidance for designing emotionally supportive and trustworthy digital mental-health systems.

The remainder of this paper is organized as follows. Section 2 describes the experimental design, including participant characteristics, the virtual doctor platform, measurement instruments, and data-analysis procedures. Section 3 presents the empirical results across subjective emotional responses, user-experience evaluations, physiological indicators, and eye-tracking measures. Section 4 discusses the findings in relation to prior literature, theoretical implications, and methodological limitations. Finally, Section 5 concludes the paper by summarizing the main contributions and outlining directions for future research.

## 2. Materials and Methods

### 2.1. Participants

Before data collection, a power analysis was conducted using G*Power 3.1 to determine the minimum required sample size. Following recommendations from Cohen and related methodological literature (Kang, 2021), a medium effect size (f = 0.25) was selected, resulting in a required minimum of 35 participants.

Data collection was conducted between March and April 2024. Participants were recruited from universities in mainland China through campus advertisements, university-affiliated social media platforms (e.g., WeChat groups), and academic personal networks. Recruitment took place over approximately six weeks, and participation was voluntary with monetary compensation provided upon completion.

The final sample comprised 42 university students aged between 19 and 27 years (M = 21.10, SD = 1.86), including 16 males (M = 23.4, SD = 3.5) and 26 females (M = 21.6, SD = 2.8). All participants reported normal or corrected-to-normal vision and basic computer literacy. None had prior experience with virtual doctors or digital mental-health interventions.

Inclusion criteria required participants to be current university students within the specified age range. Exclusion criteria included the following: (a) self-reported history of diagnosed psychiatric or neurological disorders; (b) current use of psychotropic medication; (c) cardiovascular conditions that could affect heart rate variability; and (d) excessive artifacts in physiological recordings, defined as HRV data requiring more than 10% artifact correction. Prior to participation, individuals completed a brief mental-health screening questionnaire to ensure that none had severe psychological conditions that could be adversely affected by the study.

Ethical approval for this study was obtained from the institutional ethics committee (Approval No. CCNU-IRB-202306002a). All participants provided written informed consent and were informed of the study objectives, procedures, potential risks (e.g., emotional discomfort triggered by discussing personal stress), and their right to withdraw at any time. Data were anonymized and handled in accordance with the Personal Information Protection Law (PIPL) of China.

### 2.2. Materials

#### 2.2.1. Virtual Doctor Platform

**Figure 1:**
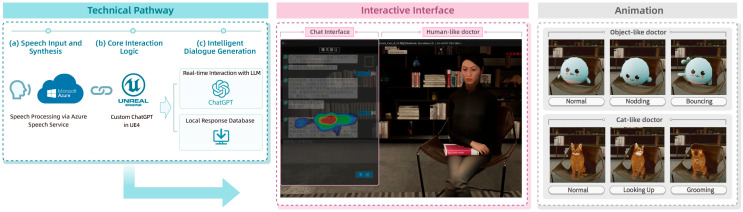
Architecture of the LLM-based virtual doctor platform. The system integrates speech recognition, large language model-based natural language generation, and text-to-speech synthesis to enable real-time interaction. User speech input is processed via speech-to-text modules and passed to the LLM, which generates contextually appropriate responses grounded in a psychological counseling framework. The generated responses are rendered as both textual and spoken output, with lip synchronization and avatar animation supporting audiovisual coherence. All three avatar embodiments (human-like, animal-like, and object-like) were equipped with idle animations consistent with their respective personas.

The virtual doctor platform was developed using Unreal Engine 4 (UE4) and OpenAI’s ChatGPT to enable natural language–based psychological consultation. As shown in Figure 1, the system integrates speech recognition, natural-language generation, and text-to-speech technologies to support seamless interaction through both voice and text (Cuadrado et al., 2020). Azure Speech Services handled speech-to-text processing, ChatGPT generated natural responses, and a text-to-speech module produced corresponding voice output. Mouth movements were synchronized with synthesized speech to enhance audiovisual coherence and user immersion (Bickmore & Picard, 2005).

Responses were generated using GPT-4 deployed through the Azure OpenAI API (API version: 2024-02-15-preview; deployment name: “gpt4-psychologist”). All interactions were conducted via the Azure API rather than the web-based interface, allowing explicit control of generation parameters and ensuring consistency across experimental conditions. Generation parameters were held constant across all avatar conditions (temperature = 0.4; top_p = 0.9; max_tokens = 200; frequency_penalty = 0.2; presence_penalty = 0.1). These settings were selected to balance response coherence with moderate variability while maintaining a counseling-appropriate tone and length constraint (approximately 100 words per response). Although all avatar conditions relied on the same Azure OpenAI deployment instance and identical generation parameters, the system response latency was not explicitly logged as a structured variable during data collection. Because response timestamps were not retained, formal statistical verification of between-condition latency equivalence is not available.

To ensure consistency across experimental conditions, all avatar embodiments relied on the same LLM (ChatGPT-4.0), identical system-level prompts, and a shared domain-specific knowledge base grounded in psychological counseling contexts. The system prompt framed the virtual doctor as a supportive mental-health professional and constrained responses to a warm yet professionally bounded tone. The prompt specified core interaction principles (emotion reflection prior to guided exploration, avoidance of prescriptive language, and provision of brief reflective summaries or small actionable suggestions), as well as ethical safeguards for crisis-related expressions. A structured summary of the system-level prompt is provided in Appendix C.

Within these constraints, dialogue generation remained open-ended and dynamically adaptive, allowing the LLM to respond flexibly to participants’ inputs during the interaction. While the thematic scope, professional role, and stylistic parameters of the virtual doctor were standardized across conditions, fine-grained linguistic variations in wording, phrasing, or emphasis could not be fully controlled, reflecting the naturalistic nature of LLM-mediated dialogue. This design choice was made to balance experimental control with ecological validity in simulated mental-health interactions.

Three avatar embodiments were created: a human-like doctor, a cat-like doctor, and an object-like doctor. Guided by the CASA framework, which suggests that people naturally attribute social characteristics to virtual agents (Nass et al., 1994; Xu et al., 2022), each avatar was designed to reflect a distinct social presence profile. The human-like avatar was modeled as an Asian female clinician to convey professionalism and warmth, consistent with the literature suggesting that human-like avatars can enhance trust in therapeutic settings (Rehm et al., 2016; Jang et al., 2025). The animal-like avatar, inspired by a Chinese rural cat, was designed to reduce anxiety and foster a relaxed atmosphere, consistent with research on the comfort-enhancing role of non-human avatars (Ichikawa et al., 2023). The object-like avatar adopted a sphere-based design with eyes and hands to retain minimal social expressiveness while providing a non-threatening and playful experience.

Avatar modeling and animation were completed using MAYA, followed by animation blueprint configuration in the Unreal Engine. Each avatar featured idle animations aligned with its persona. These behaviors included subtle book-reading animations for the human-like avatar, grooming movements for the cat avatar, and soft bouncing for the object-like avatar, enhancing social presence without introducing exaggerated or unnatural motion (Oh et al., 2018; Sinatra et al., 2021). Importantly, these idle animations were intentionally designed to be low-intensity, non-interactive, and temporally continuous across conditions, serving to maintain baseline liveliness rather than to convey task-relevant information or explicit social signals. The animation speed, amplitude, and duration were kept comparable across avatars to minimize attentional salience differences attributable to motion.

The interaction environment was designed as a quiet, warmly lit consultation room furnished with books and comfortable seating to enhance trust and emotional comfort. The literature suggests that environmental cues significantly influence emotional safety and user trust in digital mental-health settings (Reuben et al., 2022; Lawrance et al., 2022; Werkmeister et al., 2024).

#### 2.2.2. Measures

User Experience Scale: User experience was assessed using a 24-item multidimensional User Experience Scale developed to capture participants’ perceptions of the virtual doctor during the interaction. The scale comprises six dimensions, each represented by four items: Emotional Resonance, Competence, Presence, Affiliation, Appearance Evaluation, and Satisfaction. All items were rated on a five-point Likert scale ranging from 1 (strongly disagree) to 5 (strongly agree). The Emotional Resonance dimension assessed the extent to which the virtual doctor was perceived as emotionally attuned and supportive, including perceived empathy, emotional understanding, and affective engagement during the interaction. The Competence dimension captured perceived professionalism, expertise, and clarity of communication, reflecting users’ confidence in the virtual doctor’s knowledge and guidance. The Presence dimension measured perceived social and psychological presence, focusing on whether the interaction felt natural, attentive, and comparable to a real consultation. The Affiliation dimension evaluated relational warmth and approachability, including feelings of comfort, friendliness, and interpersonal connection with the virtual doctor. The Appearance Evaluation dimension focused on users’ visual impressions of the avatar, such as aesthetic appeal, appropriateness of design, and consistency between appearance and professional role. The Satisfaction dimension assessed overall evaluation of the interaction, including perceived quality, fulfillment of expectations, and willingness to engage with the virtual doctor again. The scale structure is consistent with validated frameworks commonly used in HCI and virtual agent research (Nowak & Biocca, 2003; Schuetzler et al., 2018). Items were adapted and refined to fit the specific context of LLM-based virtual doctors and were reviewed by researchers with expertise in psychology and HCI to ensure conceptual clarity and contextual relevance. This scale demonstrated excellent internal consistency in the present study (Cronbach’s α = 0.93), and each subscale showed acceptable reliability (α values ranging from 0.82 to 0.90). In the present study, the scale was used primarily for comparative analysis across avatar conditions rather than as a fully standardized psychometric instrument. The full list of scale items is provided in Appendix A, Table A1.

Uncanny Valley Questionnaire: To assess the potential uncanny effect of the human-like avatar, the Uncanny Valley Questionnaire (Mori et al., 2012) was administered only in the human-avatar condition. The 9-item scale evaluates perceived eeriness, human-likeness, and emotional distance, using a five-point rating format. Internal consistency for this scale was good (Cronbach’s α = 0.88).

Positive and negative Affect Scale (PANAS): Participants’ immediate emotional responses were measured using the Chinese version of the Positive and Negative Affect Schedule (Watson et al., 1988; Liu et al., 2020). The scale includes 20 items, comprising 10 items assessing positive affect (e.g., feelings of enthusiasm, interest, alertness, and inspiration) and 10 items assessing negative affect (e.g., distress, nervousness, irritability, and fear), rated on a five-point Likert scale. Reliability was high in this study (Positive Affect α = 0.89; Negative Affect α = 0.87).

Interaction Volume: To assess interaction volume during the free-conversation phase, we calculated the number of user utterances, system responses, and total dialogue turns for each avatar condition. A user utterance was defined as a continuous segment of participant speech, with pauses exceeding 2 s marking a new utterance. A system response was defined as each complete output generated by the virtual doctor. Total dialogue turns were computed as the sum of user utterances and system responses. All metrics were extracted from automatically logged dialogue data and verified manually. Only the free-conversation phase was included in these analyses.

Eye movements were recorded using a Tobii Fusion Pro desktop eye tracker (Tobii AB, Stockholm, Sweden) with a 120 Hz sampling rate, allowing continuous tracking of visual attention during the interaction with the virtual doctor. Based on the goals of social–cognitive analysis, two primary visual regions were defined as Areas of Interest (AOIs). The face AOI encompassed the avatar’s facial region, including the eyes, mouth, and overall facial contour, which are essential for decoding social cues, interpreting emotional expressions, and assessing interpersonal intent as noted in previous social attention research (Itier & Batty, 2009). The text AOI included the dialog text bubbles and user-input area, capturing the extent to which participants allocated attention to conversational content during the interaction. For each AOI, three commonly used gaze indicators were extracted to characterize visual-attention patterns: fixation count, total fixation duration, and time to first fixation.

Heart rate variability (HRV) was collected using a Polar H10 chest-strap device (Polar Electro Oy, Kempele, Finland) at 250 Hz across the three-minute resting baseline (T1), the entire interaction period, and an immediate post-interaction measurement (T2). HRV analysis was performed with Kubios HRV Premium with automatic artifact correction using a medium threshold, following established psychophysiological guidelines (Tarvainen et al., 2014). The time intervals between successive R-peaks (RR intervals) that exceeded physiological plausibility or deviated substantially from adjacent intervals were automatically flagged and corrected. Both time-domain and frequency-domain HRV parameters were computed. Time-domain indices included the standard deviation of normal-to-normal intervals (SDNN), reflecting overall autonomic variability, and the root mean square of successive differences (RMSSD), which is particularly sensitive to parasympathetic activity and commonly used as an index of emotional regulation and stress recovery. Frequency–domain indices included low-frequency power (LF; 0.04–0.15 Hz) and high-frequency power (HF; 0.15–0.40 Hz). HF power is primarily associated with parasympathetic modulation and respiratory sinus arrhythmia, whereas LF power reflects combined sympathetic and parasympathetic influences. The LF/HF ratio was calculated as an index of the relative autonomic balance. All HRV preprocessing and analysis followed established psychophysiological guidelines for short-term HRV measurements, including Task Force recommendations (1996) and contemporary methodological guidelines for HRV assessment in psychological research (Laborde et al., 2017).

#### 2.2.3. Experiments Procedures

**Figure 2:**
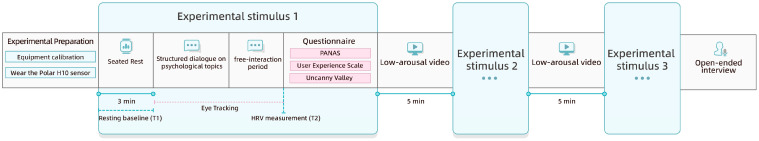
Experimental procedure. The study followed a within-subjects design in which each participant interacted with three avatar embodiments (animal-like, human-like, and object-like) in a counterbalanced order. The Uncanny Valley Questionnaire was administered only in the human-like avatar condition.

The experiment followed a within-subjects design in which each participant completed interactions with all three avatars, as illustrated in Figure 2. To minimize order effects, avatar presentation was counterbalanced using a Latin-square arrangement. Upon arrival, participants signed consent forms, completed demographic materials, completed a baseline PANAS assessment, calibrated the eye tracker, and were fitted with the HRV sensor.

Before each avatar interaction, participants underwent a three-minute resting baseline (T1). Each avatar interaction consisted of two consecutive dialogue phases. The structure and timing of both dialogue phases were identical across all avatar conditions to ensure procedural consistency. In the structured dialogue phase, participants discussed standardized psychological topics related to daily stress, emotional well-being, and coping strategies to ensure consistency in conversational content across conditions. This phase lasted approximately 10 min for each avatar. Following the structured dialogue, participants entered a free-interaction phase, during which they were instructed to engage spontaneously and naturally with the virtual doctor without predefined scripts or fixed question sets. The free-interaction phase had a minimum duration of 5 min; after this period, participants could voluntarily continue the interaction for up to an additional 5 min, resulting in a total free-interaction duration of 5–10 min per avatar. Eye-tracking and HRV signals were continuously recorded across both dialogue phases.

Immediately after each interaction, a post-interaction HRV measurement (T2) was conducted for three minutes, without any intervening rest, to capture short-term autonomic regulation following the interaction. A three-minute window was selected in accordance with established guidelines for short-term HRV assessments, which indicate that time-domain indices such as RMSSD and HF power can be reliably estimated from recordings of 2–5 min (Laborde et al., 2017).

Participants then completed the PANAS to assess post-interaction affect, along with the User Experience Scale. The Uncanny Valley Questionnaire was administered only in the human-like avatar condition. To minimize emotional carryover and physiological contamination between conditions, a neutral low-arousal video lasting approximately five minutes was presented following each avatar interaction. The video consisted of emotionally neutral nature scenes (e.g., landscapes and slow-moving scenery) without narrative or verbal content, following established affective reset procedures in psychophysiological research.

After completing all interactions, participants participated in a short open-ended interview for debriefing and contextual feedback purposes only. The interview was not intended as a qualitative dataset and was not subjected to formal qualitative analysis. The full experimental session lasted approximately ninety minutes per participant.

#### 2.2.4. Data Analysis

Data analyses were performed using SPSS 26.0 and Python 3.12.7. All variables were inspected for missing data, outliers, and distributional assumptions. The internal consistency of all questionnaire measures was assessed using Cronbach’s α. Data quality checks indicated that all HRV recordings met the predefined reliability criterion (<10% corrected RR intervals), and no participants or trials were excluded. Eye-tracking calibration quality was acceptable for all participants, and no datasets were excluded due to signal loss or missingness.

The experiment comprised a structured dialogue phase followed by a free-interaction phase for each avatar condition. Although physiological and eye-tracking data were continuously recorded throughout both phases, analyses were conducted using predefined analytic windows aligned with the theoretical aims of each measure, as described below.

Given the within-subjects design, in which each participant interacted with all three avatar conditions, repeated-measures ANOVA was used to examine the effects of the avatar type on PANAS scores, user-experience ratings, uncanny valley responses, eye-tracking variables, and HRV indicators. Greenhouse–Geisser corrections were applied when sphericity assumptions were violated, and Bonferroni-adjusted post hoc comparisons were conducted where appropriate.

Eye-tracking analyses were conducted on participant-level averaged fixation metrics within each area of interest. HRV analyses examined both post-interaction responses (T2) and within-condition change scores relative to baseline (Δ = T2 − T1).

Pearson correlations were used to explore associations among gaze metrics (e.g., proportion of face-directed fixation time and time to first fixation), HRV indicators, PANAS scores, and user-experience measures. These analyses were exploratory in nature and aimed to examine whether attentional patterns co-varied with emotional and physiological responses across conditions. All tests were two-tailed with α = 0.05.

## 3. Results

### 3.1. Effects of Avatar Type on Emotional Responses

#### 3.1.1. Subjective Emotional Responses

A repeated-measures ANOVA was conducted to examine whether the avatar type (Cat, Human, Object) influenced participants’ emotional responses. Changes in positive affect (ΔPA) did not differ significantly across the three conditions, F(2, 82) = 2.06, *p* = 0.135, η^2^p = 0.048. Likewise, changes in negative affect (ΔNA) showed no significant effect of the avatar type, F(2, 82) = 0.42, *p* = 0.658, η^2^p = 0.010.

Descriptive statistics indicated small mean changes in affect across all avatar conditions. For positive affect, mean ΔPA scores were 0.14 (SD = 0.86) for the Cat avatar, 0.30 (SD = 0.75) for the Human avatar, and 0.16 (SD = 0.61) for the Object avatar. For negative affect, mean ΔNA scores were 0.47 (SD = 0.53), 0.54 (SD = 0.59), and 0.48 (SD = 0.56) for the Cat, Human, and Object avatars, respectively.

**Figure 3:**
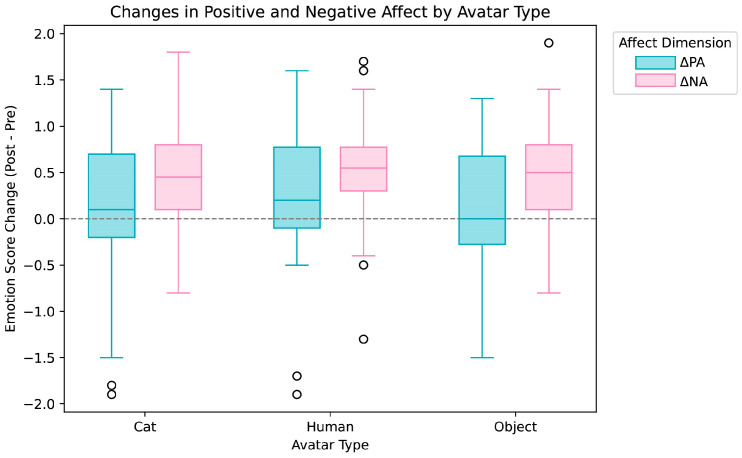
Boxplots of changes in positive affect (ΔPA) and negative affect (ΔNA) across the three avatar conditions (Cat, Human, Object). Circles represent outliers beyond 1.5× the interquartile range.

As shown in Figure 3, distributions of ΔPA and ΔNA were largely overlapping across the Cat, Human, and Object avatars. Accordingly, the avatar type did not yield statistically significant differences in changes in positive or negative affect, indicating that medium or larger effects were not detected under the present experimental conditions.

#### 3.1.2. Physiological Responses

**Figure 4:**
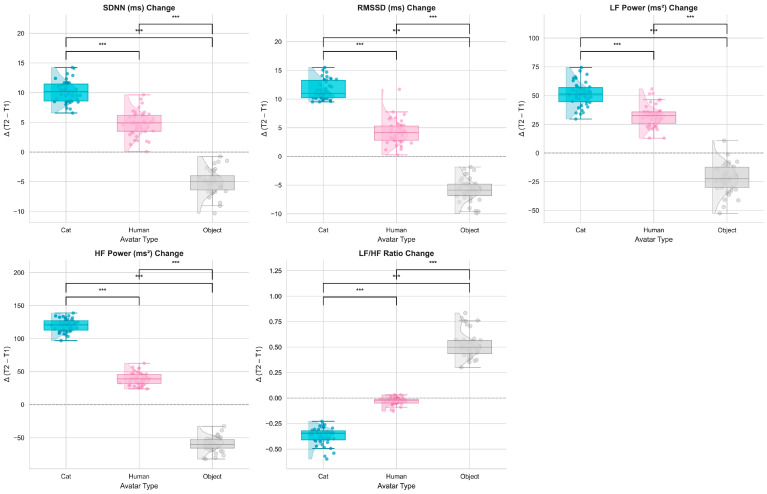
Box plots and scatter plots showing HRV changes (mean ± SE) for SDNN, RMSSD, LF, HF, and LF/HF ratio from baseline (T1) to post-interaction (T2) across three avatar types (Cat, Human, Object). Repeated-measures ANOVA revealed significant differences between avatar conditions, with the Cat avatar eliciting the highest HRV responses. Asterisks indicate significant pairwise differences (*** *p* < 0.001).

To assess whether avatar embodiment influenced autonomic activity, heart rate variability (HRV) indices were compared between the baseline (T1) and the immediate post-interaction period (T2) across the three avatar conditions. Significant effects of avatar type emerged across multiple HRV metrics (Figure 4).

For SDNN, a general index of overall autonomic variability, a significant main effect of avatar type was observed, F(2, 82) = 11.92, *p* < 0.001, η^2^ = 0.33. SDNN increased notably in both the Cat and Human conditions, with the Cat avatar producing the largest increase, whereas the Object avatar elicited a decrease. Post-hoc comparisons indicated that SDNN was significantly higher in the Cat condition than in both the Human (*p* = 0.001) and Object (*p* < 0.001) conditions, suggesting a more relaxed physiological state during interactions with the Cat avatar.

RMSSD, a parasympathetic indicator, also revealed a significant effect of the avatar type, F(2, 82) = 13.56, *p* < 0.001, η^2^ = 0.36. RMSSD increased substantially in the Cat condition, whereas it showed only minimal change in the Human condition and a slight decrease in the Object condition. Post-hoc tests confirmed significantly higher RMSSD responses in the Cat condition relative to the Human (*p* = 0.001) and Object (*p* < 0.001) conditions.

LF power, reflecting sympathetic activation, varied significantly across avatar types, F(2, 82) = 15.47, *p* < 0.001, η^2^ = 0.38. LF gains were greatest under the Cat condition, significantly exceeding those in the Human (*p* = 0.001) and Object (*p* < 0.001) conditions. These increases suggest heightened—but non-stressful—arousal, consistent with adaptive engagement.

HF power, a parasympathetic index, also differed significantly among avatar types, F(2, 82) = 13.23, *p* < 0.001, η^2^ = 0.35. HF increased most strongly in the Cat condition but declined slightly in the Object condition. Post-hoc comparisons indicated that HF was significantly higher in the Cat than in both the Human (*p* = 0.002) and Object (*p* = 0.001) conditions.

For the LF/HF ratio, an index of the sympathetic–parasympathetic balance, a significant effect of avatar type emerged, F(2, 82) = 10.02, *p* < 0.001, η^2^ = 0.29. The Object condition produced the highest LF/HF ratio, indicating a sympathetic-dominant response and suggesting elevated stress. Significant differences were observed between Cat vs. Object (*p* = 0.001) and Human vs. Object (*p* = 0.001).

**Figure 5:**
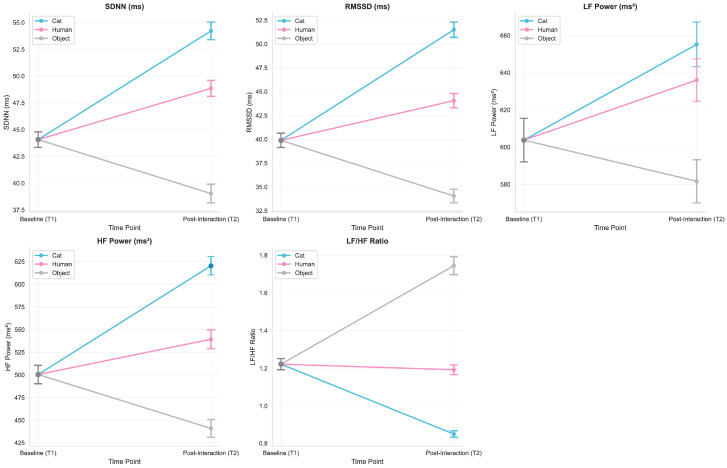
Line plots showing HRV metrics (mean ± SE) at baseline (T1) and post-interaction (T2) across three avatar types (Cat, Human, Object). Repeated-measures ANOVA revealed significant changes over time, with the Cat avatar showing the most pronounced HRV changes.

Further inspection of temporal changes (Figure 5) revealed distinct autonomic response patterns. The Cat avatar elicited robust increases in both parasympathetic (RMSSD, HF) and sympathetic (LF) activity from T1 to T2, reflecting a relaxed but engaged physiological profile. The Human avatar produced a mixed pattern, with increases in LF but modest reductions in RMSSD. In contrast, the Object avatar induced minimal HRV changes and an elevated LF/HF ratio, indicating comparatively higher physiological stress.

**Figure 6:**
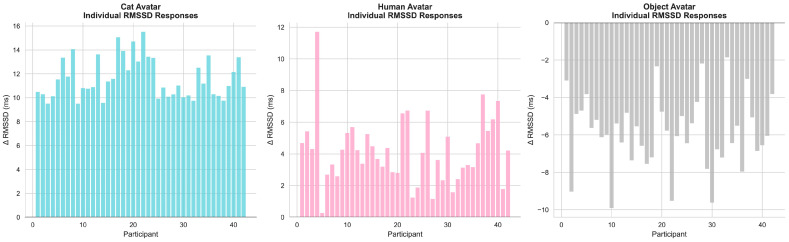
Within-subject RMSSD change scores (ΔRMSSD = T2 − T1) across avatar conditions. Bars represent individual participants’ change scores and are shown for descriptive purposes only. Group-level effects were tested using repeated-measures ANOVA.

Descriptive distributions of within-subject RMSSD change scores are shown in Figure 6. The visualization illustrates that RMSSD change values tended to be positive in the Cat avatar condition, whereas more negative change values were observed in the Object avatar condition. These distributions provide a descriptive overview of inter-individual variability in autonomic responses, while inferential conclusions are drawn from group-level statistical analyses.

#### 3.1.3. Interaction Volume Across Conditions

To examine whether the interaction volume differed across avatar conditions, repeated-measures ANOVAs were conducted for the free-conversation duration, total word count, total dialogue turns, number of user utterances, and number of system responses.

Although the animal-like avatar showed slightly higher descriptive values across most indicators, no statistically significant main effects of avatar type were observed for any interaction-volume measure (all *p* > 0.05; see Table A2). Effect sizes were small, indicating minimal between-condition variation in the conversation length or turn-taking structure.

### 3.2. Effects of Avatar Type on User Experience

**Figure 7:**
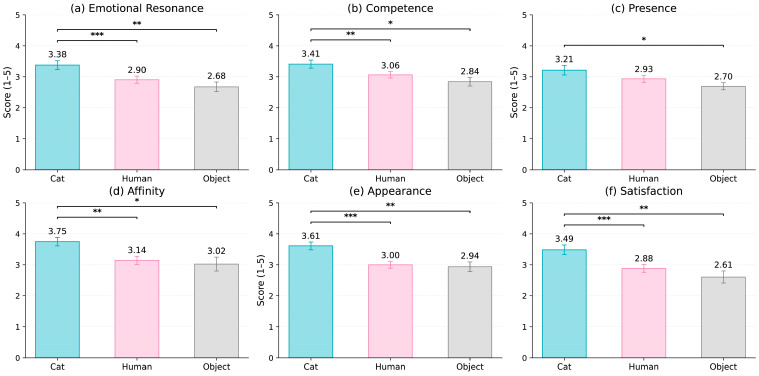
Comparison of user-experience ratings (mean ± SE) across six evaluative dimensions for the Cat, Human, and Object avatars. Repeated-measures ANOVA revealed consistent effects of the avatar type, with the Cat avatar rated highest based on all dimensions. Asterisks denote significant pairwise differences (* *p* < 0.05, ** *p* < 0.01, *** *p* < 0.001).

A series of repeated-measures ANOVAs was conducted to evaluate whether the avatar type (Cat, Human, Object) influenced participants’ evaluations across six user-experience dimensions: Emotional Resonance, Competence, Presence, Affiliation, Appearance Evaluation, and Satisfaction. As shown in Figure 7, consistent and robust effects of the avatar type were observed across all dimensions. Unless otherwise noted, all F statistics reflect uncorrected degrees of freedom; the application of Greenhouse–Geisser corrections did not alter the significance patterns.

For Emotional Resonance, the avatar type exerted a significant main effect, F(2, 82) = 9.34, *p* = 0.0002, partial η^2^ = 0.19. Participants reported the highest emotional resonance with the Cat avatar (M = 3.38, SE = 0.14), followed by the Human avatar (M = 2.90, SE = 0.12), and the lowest ratings for the Object avatar (M = 2.68, SE = 0.15).

A similar pattern emerged for Competence, F(2, 82) = 6.58, *p* = 0.0022, partial η^2^ = 0.14, with the Cat avatar rated as most competent (M = 3.41, SE = 0.13), followed by the Human (M = 3.06, SE = 0.11) and Object (M = 2.84, SE = 0.14) avatars.

For Presence, the main effect of avatar type was significant, F(2, 82) = 5.32, *p* = 0.0067, partial η^2^ = 0.11. Presence ratings were highest for the Cat avatar (M = 3.21, SE = 0.15), moderate for the Human avatar (M = 2.93, SE = 0.14), and lowest for the Object avatar (M = 2.70, SE = 0.15).

The same directional trend was observed for Affiliation, F(2, 82) = 4.76, *p* = 0.0110, partial η^2^ = 0.10. Participants rated the Cat avatar highest in warmth and interpersonal affinity (M = 3.75, SE = 0.14), followed by the Human avatar (M = 3.14, SE = 0.14) and the Object avatar (M = 3.02, SE = 0.23).

For the Appearance Evaluation, the avatar type again yielded a significant effect, F(2, 82) = 8.47, *p* = 0.00045, partial η^2^ = 0.17. Participants rated the Cat avatar as the most visually appealing (M = 3.61, SE = 0.12), with substantially lower ratings for the Human (M = 3.00, SE = 0.11) and Object avatars (M = 2.94, SE = 0.16).

Finally, Satisfaction differed significantly across avatar types, F(2, 82) = 8.70, *p* = 0.00037, partial η^2^ = 0.18. Satisfaction was highest in the Cat condition (M = 3.49, SE = 0.15), followed by the Human (M = 2.88, SE = 0.13) and Object conditions (M = 2.61, SE = 0.19).

Across all six user-experience dimensions, a highly consistent pattern emerged: Cat > Human > Object, with moderate-to-large effect sizes (partial η^2^ approximately 0.10–0.19). These results provide strong support for the hypothesis that animal-like avatars elicit more positive subjective experiences across emotional, social, and aesthetic domains compared to human-like and object-like avatars.

### 3.3. Uncanny Valley Effect in Human-like Virtual Doctors

To assess whether the human-like virtual doctor elicited an uncanny valley response, we examined the associations between three uncanny valley indicators—Eeriness, Human-likeness, and Expressiveness—and participants’ user-experience ratings. Descriptive statistics indicated moderate levels of eeriness (M = 3.14), relatively low human-likeness (M = 2.87), and moderate expressiveness (M = 3.31).

**Figure 8:**
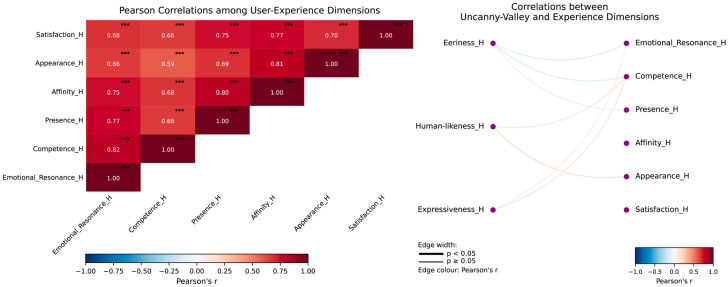
Correlation heatmap between uncanny valley indicators and user-experience dimensions for the human-like avatar. *** *p* < 0.001.

Correlation analyses revealed only weak and nonsignificant associations between uncanny valley responses and user-experience outcomes (Figure 8). Eeriness showed small negative correlations with Emotional Resonance (r = −0.19), Competence (r = −0.19), and Presence (r = −0.11), whereas Human-likeness and Expressiveness showed small positive correlations with Competence and Appearance (0.10 < r < 0.23). None of these correlations reached statistical significance (all *p* > 0.23).

Regression analyses further tested whether uncanny valley indicators predicted user experience. Across all models, standardized regression coefficients were small (|β| < 0.23), and confidence intervals consistently crossed zero. Human-likeness showed a modest but nonsignificant positive trend in predicting Appearance (β = 0.228, *p* = 0.146), whereas Eeriness showed nonsignificant negative trends for Emotional Resonance (β = −0.188, *p* = 0.234) and Competence (β = −0.189, *p* = 0.231).

Taken together, the results provide no strong evidence that the human-like virtual doctor elicited an uncanny valley effect. Although some effects were directionally consistent with uncanny valley theory—such as greater eeriness predicting lower social evaluations—the magnitudes were small, and none reached statistical significance. Overall, the human-like avatar may not have reached the realism threshold necessary to trigger a pronounced uncanny valley response.

### 3.4. The Impact of Visual Imagery on User Attention

**Figure 9:**
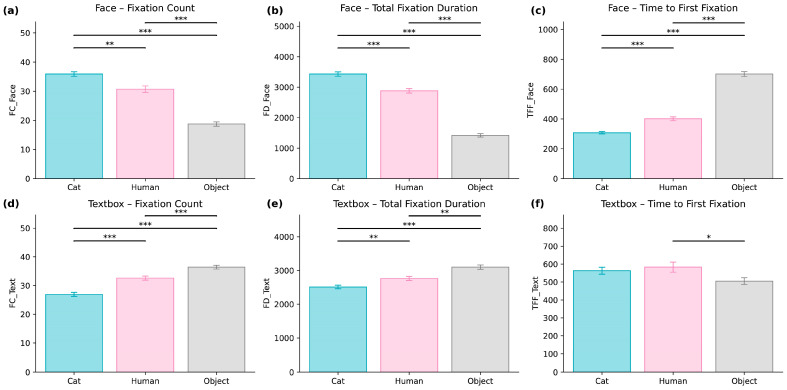
Eye-tracking metrics across avatar conditions for Face and Textbox AOIs. (**a**–**f**) Mean values (±s.e.m.) of fixation count, total fixation duration, and time to first fixation (TFF) for Cat, Human, and Object avatars across two areas of interest (AOI-Face and AOI-Textbox). Significant pairwise differences were assessed using Bonferroni-corrected paired *t*-tests, with asterisks indicating significance (* *p* < 0.05; ** *p* < 0.01; *** *p* < 0.001). Error bars represent the standard error of the mean.

To evaluate how avatar appearance influenced users’ attentional allocation during the interaction, we analyzed eye-tracking data across the three avatar types, namely Cat (animal-like), Human (human-like), and Object (object-like). Attention was examined within two Areas of Interest (AOIs), which were the facial region (Face AOI) and the dialog text region (Textbox AOI). The results demonstrated clear and systematic effects of the avatar visual design on users’ gaze behavior, as presented in Table 2 and Figure 9a–f.

Face AOIs were defined individually for each avatar based on visible facial boundaries, including the eyes, mouth, and overall facial contour. Although avatar morphology differed across conditions, AOIs were constructed using consistent anatomical criteria. Because face sizes varied across embodiments, AOI pixel areas were not identical; exact dimensions and screen-percentage coverage are reported in Table A3. To mitigate potential confounds related to the interaction duration or AOI size, fixation-based indices were examined both in absolute terms and as proportion-based metrics (e.g., proportion of total fixation time allocated to the Face AOI). These normalization procedures ensure that observed differences in gaze behavior more accurately reflect attentional allocation rather than structural differences in the AOI size.

For the Face AOI, significant main effects of avatar type were found for all facial attention metrics. These included the fixation count, F(2, 82) = 86.87, *p* = 5.58 × 10^−21^, η^2^p = 0.609; total fixation duration, F(2, 82) = 249.23, *p* = 1.42 × 10^−35^, η^2^p = 0.789; and time to first fixation (TFF), F(2, 82) = 235.01, *p* = 1.11 × 10^−34^, η^2^p = 0.803. Post-hoc comparisons (Table 2) indicated that the Cat avatar received significantly more fixations and longer viewing durations than both the Human and Object avatars, with all *p* values below 0.001. The Object avatar consistently received the lowest level of facial attention.

To further account for the AOI size and interaction duration, supplementary analyses were conducted using normalized gaze indices. Specifically, we calculated (a) the proportion of the fixation duration allocated to the Face AOI relative to the total fixation duration (Face + Text) and (b) the proportion of the fixation count allocated to the Face AOI relative to total fixation count. For the fixation-duration proportion, a repeated-measures ANOVA revealed a significant main effect of avatar type, F(2, 82) = 258.44, *p* < 0.001, η^2^p = 0.863. The Cat avatar (M = 0.577, SD = 0.050) elicited the highest proportion of the face-directed fixation time, followed by the Human avatar (M = 0.509, SD = 0.053) and the Object avatar (M = 0.312, SD = 0.065). A similar pattern emerged for the fixation-count proportion, F(2, 82) = 152.20, *p* < 0.001, η^2^p = 0.788, with the Cat avatar (M = 0.571, SD = 0.051) receiving the greatest proportion of face-directed fixations, followed by the Human (M = 0.480, SD = 0.072) and Object avatars (M = 0.337, SD = 0.061). Pairwise comparisons for normalized metrics are also reported in Table 2. Importantly, the normalized results mirror the raw fixation findings, indicating that embodiment effects were not attributable to AOI size differences or the interaction duration.

**Figure A1:**
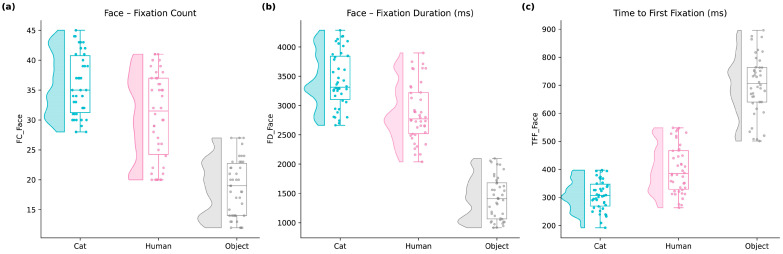
Distribution of Face AOI attention across avatar conditions. (**a**–**c**) Raincloud plots showing the distribution, kernel density estimates, and individual data points for fixation count, total fixation duration, and time to first fixation on the avatar’s face. Cat, Human, and Object avatar conditions are visualized using violin, box, and jittered point components. All data represent repeated-measures observations.

**Figure A2:**
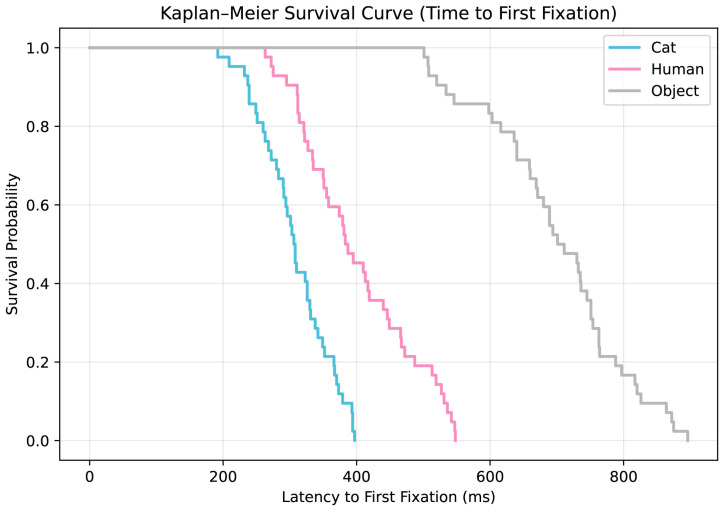
Kaplan–Meier survival curves for time to first fixation on the avatar’s face. Survival curves represent the probability that participants had not yet fixated on the avatar’s face over time during the interaction task. Separate curves are shown for Cat, Human, and Object avatars. Curves were estimated using the Kaplan–Meier method, with shaded bands indicating 95% confidence intervals. A steeper decline indicates faster initial visual engagement.

Although the Human avatar attracted more facial fixations than the Object avatar, it was associated with a longer TFF compared with the Cat avatar, indicating delayed initial orientation toward the facial region. Raincloud plots (Appendix B, Figure A1) illustrate the separation of attention distributions across avatar conditions, with the Cat avatar showing the most concentrated facial-attention pattern and the Human and Object avatars exhibiting greater variability. Kaplan–Meier survival curves (Figure A2) further show that participants oriented toward the Cat avatar’s face most rapidly, followed by the Human avatar, while the Object avatar displayed the slowest orientation times.

The avatar type also exerted a significant influence on attention directed toward the text region. The fixation count differed significantly across conditions, F(2, 82) = 55.15, *p* = 6.67 × 10^−16^, η^2^p = 0.440, as did the total fixation duration, F(2, 82) = 24.40, *p* = 4.85 × 10^−9^, η^2^p = 0.286. Participants devoted more visual attention to the text region when interacting with the Cat avatar compared with both the Human and Object avatars, with all *p* values below 0.01.

For TFF in the Textbox AOI, a smaller but significant effect of the avatar type was observed, F(2, 82) = 3.33, *p* = 0.0408, η^2^p = 0.050. Post hoc comparisons indicated that participants oriented toward the text region more rapidly in the Object avatar condition than in the Cat avatar condition (*p* = 0.016).

Across both AOIs, the Cat avatar consistently elicited the strongest and most rapid attentional engagement. The Human avatar produced moderate levels of facial engagement but showed delayed initial attention, consistent with subtle delayed initial engagement patterns. The Object avatar drew the least social attention and prompted the quickest transition toward text-based information, reflecting its low level of social cue richness.

## 4. Discussion

The present study integrated subjective affect ratings, user-experience evaluations, physiological measures of autonomic activity, and eye-tracking indicators to investigate how different virtual doctor embodiments shape users’ emotional, cognitive, and attentional responses during LLM-based mental-health interactions. Across these multimodal measures, two central observations emerged. First, avatar appearance exerted a systematic influence on social processing and attention allocation, although emotional self-reports and physiological reactions exhibited partially dissociated patterns. Second, the animal-like avatar consistently produced more favorable subjective and objective outcomes, whereas the human-like avatar elicited moderate social engagement without clear evidence of a strong uncanny valley response. Together, these findings suggest that visual embodiment may influence user experience through multiple, partially independent pathways, rather than through a single unified affective mechanism. This constellation of findings refines existing theoretical perspectives of human–agent interactions by suggesting that emotional safety and socio-affiliative affordances play a more decisive role than anthropomorphism alone in shaping responses to virtual clinicians.

### 4.1. Multimodal Effects of Avatar Embodiment

A key contribution of this study concerns the divergence between subjective emotional reports and physiological responses. From a statistical perspective, the non-significant PANAS findings indicate that medium or larger effects of avatar embodiment on explicit affective reports were not observed, although smaller effects cannot be excluded. At the physiological level, interactions with the animal-like avatar were associated with higher HRV indices, including RMSSD and HF power, whereas the object-like avatar showed comparatively lower HRV values. These patterns suggest potential differences in autonomic regulation across avatar conditions during the interaction.

It is important to note that HRV indices reflect integrated autonomic activity during ongoing cognitive and communicative tasks, rather than isolated emotional states. Factors such as the cognitive load, respiration, posture, and speech-related activity are inherent to interactive dialogue and may contribute to observed variability in both time- and frequency-domain measures (Laborde et al., 2017). Accordingly, indices such as LF, HF, and LF/HF are interpreted here as indicators of task-related autonomic modulation, rather than as direct or unitary markers of stress or affective valence.

Within this context, the dissociation between self-reported affect and physiological responses aligns with dual-systems accounts suggesting that conscious emotional experience and autonomic regulation may operate partially independently (Mauss & Robinson, 2009). Research on implicit emotional processing further indicates that autonomic and attentional responses can reflect affective appraisal processes that occur outside conscious awareness and may not be readily captured by self-report instruments (Hoemann et al., 2021). In such contexts, physiological measures may be more sensitive to subtle variations in perceived emotional safety, comfort, or threat than explicit affect ratings.

This divergence may be further accentuated by the cognitively demanding nature of LLM-mediated interactions. Participants in the present study engaged in real-time language comprehension, response planning, and conversational turn-taking, all of which impose a nontrivial cognitive load. Under such conditions, individuals may show attenuated or less differentiated self-reported emotional responses, even when underlying physiological modulation is present. Accordingly, while conscious affective awareness may have been dampened during the interaction, autonomic indices continued to reflect subtle variations in emotional regulation associated with avatar appearance (Shaffer & Ginsberg, 2017). These findings underscore the importance of incorporating psychophysiological measures when evaluating emotional experience in digitally mediated mental-health contexts.

User-experience evaluations further demonstrated a robust and consistent pattern: the animal-like avatar was rated highest across emotional resonance, presence, appearance, and satisfaction, followed by the human-like avatar, with the object-like avatar receiving the lowest evaluations. While this pattern partially aligns with CASA predictions that more socially expressive agents elicit stronger social responses (Ho et al., 2018; Klein, 2025), the superior performance of the animal-like avatar suggests that anthropomorphism alone does not determine user experience. Instead, these results resonate with research on cross-species social affinity, which suggests that non-threatening animals can evoke strong feelings of safety, warmth, and social openness (Premathilake & Li, 2024). Such affective affordances may enhance emotional resonance and reduce social defensiveness—a desirable characteristic for mental-health applications. Consistent with findings from human–animal interaction literature (Beetz et al., 2012; Ahmed et al., 2024). Taken together, these results tentatively suggest that perceived emotional safety may outweigh human resemblance as a determinant of user comfort in virtual mental-health interactions.

By contrast, although the human-like avatar contained clear social cues, it did not provide experiential advantages over the animal-like design. Its moderate realism may not have been sufficient to activate human-like social schemas fully (S. Schneider et al., 2022; Dubois-Sage et al., 2023), while limitations in facial expressiveness or micro-behavioral dynamics may have introduced subtle inconsistencies. These features could result in mild perceptual discomfort, consistent with research showing that mid-realism avatars often lack the expressive coherence required to sustain trust and comfort (Baake et al., 2025; Huang et al., 2025).

At the same time, theoretical accounts of the uncanny valley suggest that uncanny responses can manifest at multiple processing levels, ranging from early perceptual or autonomic reactions to consciously accessible evaluations (Mauss & Robinson, 2009; Cheetham, 2011). As a result, more subtle or preconscious forms of perceptual unease may not necessarily be reflected in explicit self-report measures alone, particularly in cognitively engaging interaction contexts (Hoemann et al., 2021).

However, it is notable that the human-like avatar in this study did not elicit a strong uncanny valley effect. Correlation and regression analyses showed no significant relationships among eeriness, human-likeness, and user-experience ratings, suggesting that the avatar did not surpass the “realism threshold” required to evoke intense uncanny sensations (Mori et al., 2012; Thaler et al., 2021). Nevertheless, the delayed first fixation toward the human-like avatar’s face indicates subtle early perceptual hesitation, consistent with prior findings showing that delayed initial facial engagement may occur prior to conscious reports of eeriness (Cheetham, 2011). Importantly, this delayed initial engagement effect was mild and did not translate into negative experiential evaluations.

Moreover, supplementary analyses indicated no significant between-condition differences in the interaction volume, suggesting that the observed embodiment effects are unlikely to be attributable to variations in the conversation duration or turn-taking structure, although unmeasured temporal factors (e.g., response latency) cannot be entirely ruled out.

### 4.2. The Impact of Visual Imagery on User Attention

Eye-tracking findings revealed that the avatar appearance systematically influenced attention allocation. The animal-like avatar attracted greater face-directed attention, reflected in more fixations, longer viewing durations, and faster initial orientation, consistent with theories of social attention suggesting that affiliative facial features preferentially capture visual processing (End & Gamer, 2017; Kawakami et al., 2024).

Importantly, this pattern remained robust when normalized gaze metrics were considered. Even after accounting for differences in the AOI size and interaction duration, the animal-like avatar elicited the highest proportion of the face-directed fixation time and fixation count, whereas the object-like avatar received the lowest proportional allocation of facial attention. This consistency indicates that the observed embodiment effects reflect genuine differences in attentional prioritization rather than structural characteristics of the stimulus display.

The human-like avatar elicited moderate but less immediate facial attention, indicating delayed initial engagement relative to the animal-like avatar. This pattern may reflect more cautious processing of near-human features. In contrast, the object-like avatar received minimal facial attention and prompted faster shifts toward text-based information, consistent with attention-allocation patterns observed in interactions with reduced social salience. Taken together, these findings suggest that avatar embodiment may shape not only users’ affective impressions but also their attentional strategies during the interaction, suggesting that attentional allocation may represent a potential correlate of experiential and physiological variation across embodiment conditions.

### 4.3. Limitations and Future Directions

Several limitations of the present study should be acknowledged. First, although the sample size was appropriate for the experimental design, participants were predominantly young university students without clinical diagnoses, limiting generalizability to clinical populations and precluding direct claims regarding therapeutic effectiveness.

Second, it is also possible that the PANAS, as a broad measure of general positive and negative affect, may not have been sufficiently sensitive to capture subtle, interaction-specific variations in emotional experience elicited by different avatar embodiments. Future studies may benefit from incorporating more fine-grained mood instruments or domain-specific measures—such as scales assessing perceived emotional safety, interpersonal comfort, or social presence—that are more directly aligned with the socio-relational dynamics of human–avatar interactions.

Third, perceptions related to the uncanny valley were assessed only within the human-like avatar condition, which limits the ability to conduct direct between-condition comparisons of eeriness, human-likeness, or familiarity across avatar types. This design choice reflects the theoretical origin of the uncanny valley construct, which primarily concerns near-human agents; however, it precludes the use of these measures as a full manipulation check across all embodiments. Consequently, conclusions regarding the relative degree of uncanniness should be interpreted with caution. Future studies may benefit from administering comparable perceptual measures across all avatar conditions to more systematically validate embodiment-related differences.

In addition, system response latency was not explicitly recorded or modeled as a formal analytic variable. Although all avatar conditions relied on the same backend architecture, API deployment, and generation parameters, unmeasured variation in response latency cannot be fully excluded. Differences in waiting time may influence perceived responsiveness, cognitive load, or attentional dynamics during the interaction. Future studies should log and statistically evaluate response latency to more precisely disentangle embodiment effects from potential temporal factors in LLM-mediated dialogue.

Finally, although avatar embodiments were primarily differentiated by visual appearance, each avatar also featured subtle idle animations aligned with its persona. While designed to be low-intensity, these animations may have contributed marginally to differences in visual attention and engagement. Future work could further disentangle the effects of appearance and motion by systematically controlling or manipulating animation features across avatar types.

### 4.4. Summary and Implications

Taken together, the multimodal findings indicate that the virtual doctor appearance may shape user experience through interconnected emotional, cognitive, and physiological pathways. Importantly, these findings should be interpreted as experimental and exploratory evidence derived from a controlled laboratory setting, rather than as demonstrations of direct clinical effectiveness. The results extend CASA and uncanny valley frameworks by demonstrating that emotional safety, affiliative design cues, and perceptual fluency are critical moderators of human–agent interactions, particularly in mental-health settings. From an applied perspective, the present findings should be interpreted as providing design-oriented insights rather than direct clinical recommendations. The consistent advantages of the animal-like avatar highlight its potential relevance in low-stakes or preliminary interaction contexts, such as early engagement or supportive check-ins, rather than established clinical interventions.

From an exploratory and translational perspective, these findings offer preliminary guidance for the design of virtual mental-health systems. Rather than implying immediate clinical deployment, the observed advantages of animal-like embodiments suggest that such designs may be promising candidates for further investigation in contexts where emotional comfort and approachability are prioritized, such as experimental triage interfaces, youth-oriented support tools, or early-stage digital mental-health services. Human-like embodiments may be better suited for psychoeducation or structured guidance provided that expressive realism and behavioral fidelity are sufficiently advanced, while object-like embodiments may be appropriate for task-focused or informational use cases.

Crucially, the present results do not substitute for clinical validation. Future work should examine how these design principles generalize across cultures, age groups, and clinically diagnosed populations, and whether repeated or long-term exposure to different avatar embodiments influences therapeutic alliance, adherence, or emotional resilience under real-world conditions.

## 5. Conclusions

The present study examined how three types of virtual doctor embodiments influence users’ emotional reactions, physiological regulation, user experience, and visual attention during mental health interactions. By integrating subjective reports, physiological signals, and gaze behavior, the findings suggest that avatar visual embodiment is associated with measurable and systematic differences in users’ experiential, autonomic, and attentional responses.

Across multiple indicators, the animal-like avatar produced the most favorable interaction outcomes. It was associated with more positive user experience evaluations, stronger autonomic regulation, and greater social attention. The human-like avatar elicited moderate engagement without clear evidence of pronounced uncanny valley effects, while the object-like avatar consistently produced lower levels of emotional involvement and social attention. Taken together, these results underscore the potential importance of emotional safety, perceived warmth, and social clarity in shaping user engagement with virtual clinicians, beyond anthropomorphism alone.

The findings further highlight the methodological value of a multimodal assessment approach for digital mental-health research. Physiological and attentional indicators were sensitive to differences in avatar embodiment even when self-reported affective measures showed no significant variation, suggesting that implicit and behavioral measures can capture aspects of user experience that may not be accessible through explicit self-report alone, particularly in cognitively demanding interaction scenarios.

Several limitations should be considered when interpreting these findings. The study was conducted with a non-clinical sample of young university students in a controlled laboratory setting and focused on short-term interactions, which limits generalizability to clinical populations and long-term therapeutic contexts. In addition, although the linguistic style and emotional tone of the LLM-generated responses were constrained, fine-grained variations in dialogue content were unavoidable. Accordingly, the present results should be interpreted as experimental and exploratory rather than as evidence of direct clinical effectiveness.

Future research should extend this work by examining avatar embodiment effects across diverse age groups, cultural backgrounds, and clinically diagnosed populations, as well as through longitudinal designs that assess sustained engagement, trust formation, and therapeutic alliance over time. Further investigation into the interaction between visual embodiment, verbal strategies, and adaptive behavioral cues will be essential for informing the evidence-based design of virtual clinicians in digital mental-health applications.

## Figures and Tables

**Table 1:** Representative studies on avatar embodiment in healthcare and related contexts.

Study	Application Context	Avatar Type	Key Measures	Main Findings	Limitations
(Kätsyri et al., 2015)	Virtual character perception	Human-like (varying realism)	Self-report, enaffect	Near-human realism increased eeriness	Not healthcare-specific
(Rehm et al., 2016)	Virtual health assistants	Human-like	Trust, perceived competence	Human-like appearance enhanced credibility	No comparison with non-human avatars
(Thaler et al., 2021)	Social interaction with virtual agents	Human-like (varying realism)	Eye tracking, self-report	Near-human avatars elicited delayed initial engagement patterns consistent with uncanny valley	Not focused on healthcare
(Ichikawa et al., 2023)	Companion agents	Animal-like	Anxiety, comfort	Animal avatars reduced anxiety and promoted relaxation	Single avatar category
(Lim et al., 2024)	Emotional support agents	Animal-like vs. human-like	User experience	Animal-like agents perceived as warmer and less threatening	No physiological indicators
(Llanes-Jurado et al., 2024).	Human–robot interaction	Mixed embodiments	HRV, stress indicators	Agent appearance modulated autonomic responses	No direct avatar-type comparison
(Mazerant et al., 2025)	Functional AI agents	Object-like	Task performance, attention	Object-like designs reduced social engagement	Not applied to mental health

**Table 2:** Bonferroni-corrected pairwise comparisons between avatar types for Face AOI and Text AOI measures.

	**Face Fixation Count**			**Textbox Fixation Count**		
Comparison	T (df)	*p* (Bonf.)	Hedges’ g	T (df)	*p* (Bonf.)	Hedges’ g
Cat vs. Human	3.54 (41)	0.003	0.815	5.17 (41)	*p* < 0.001	0.771
Cat vs. Object	14.95 (41)	*p* < 0.001	3.442	9.15 (41)	*p* < 0.001	1.708
Human vs. Object	8.74 (41)	*p* < 0.001	1.932	4.38 (41)	*p* < 0.001	0.711
	**Face Fixation Duration**			**Textbox Fixation Duration**		
Cat vs. Human	6.74 (41)	*p* < 0.001	1.133	3.37 (41)	0.004	0.617
Cat vs. Object	19.85 (41)	*p* < 0.001	4.738	7.64 (41)	*p* < 0.001	1.436
Human vs. Object	13.11 (41)	*p* < 0.001	2.412	4.27 (41)	*p* < 0.001	0.805
	**Time to First Fixation (Face)**			**Time to First Fixation (Textbox)**		
Cat vs. Human	−6.80 (41)	*p* < 0.001	−1.134	1.20 (41)	0.694	0.209
Cat vs. Object	−22.18 (41)	*p* < 0.001	−4.857	−2.87 (41)	0.017	−0.461
Human vs. Object	−10.91 (41)	*p* < 0.001	−2.008	−1.76 (41)	0.264	−0.289
	**Face Fixation Proportion (Duration-Based)**			**Face Fixation Proportion (Count-Based)**		
Cat vs. Human	6.63 (41)	*p* < 0.001	1.10	5.74 (41)	*p* < 0.001	0.95
Cat vs. Object	19.78 (41)	*p* < 0.001	4.72	14.47 (41)	*p* < 0.001	3.36
Human vs. Object	13.08 (41)	*p* < 0.001	2.40	7.66 (41)	*p* < 0.001	1.78

## Data Availability

The original contributions presented in this study are included in the article. Further inquiries can be directed to the corresponding author.

## Abbreviations

The following abbreviations are used in this manuscript:

AOI	Area of Interest
CASA	Computers as Social Actors
HF	High-Frequency Power
HCI	Human–Computer Interaction
HRV	Heart Rate Variability
LF	Low-Frequency Power
LLM	Large Language Model
PANAS	Positive and Negative Affect Schedule
RMSSD	Root Mean Square of Successive Differences
SDNN	Standard Deviation of Normal-to-Normal Intervals

## Appendix A

**Table A1:** User Experience Scale Items.

**A.1. Emotional Resonance**
ER1. The virtual doctor understood my emotional state.
ER2. I felt emotionally supported during the interaction.
ER3. The virtual doctor responded appropriately to my feelings.
ER4. The interaction felt emotionally engaging.
**A.2. Competence**
C1. The virtual doctor appeared knowledgeable.
C2. The guidance provided was reasonable and helpful.
C3. The virtual doctor communicated clearly.
C4. The virtual doctor behaved professionally.
**A.3. Presence**
P1. I felt that the virtual doctor was truly present during the interaction.
P2. The interaction felt natural.
P3. The virtual doctor seemed attentive to me.
P4. The interaction resembled a real consultation.
**A.4. Affiliation**
A1. I felt comfortable interacting with the virtual doctor.
A2. The virtual doctor felt approachable.
A3. I felt a sense of connection with the virtual doctor.
A4. The interaction felt friendly.
**A.5. Appearance Evaluation**
AE1. The appearance of the virtual doctor was visually appealing.
AE2. The avatar design felt appropriate for its role.
AE3. The visual style of the avatar was pleasant.
AE4. The avatar’s appearance matched its professional function.
**A.6. Satisfaction**
S1. I was satisfied with the interaction overall.
S2. I would be willing to interact with this virtual doctor again.
S3. The experience met my expectations.
S4. Overall, the interaction was positive.

**Table A2:** Summary statistics of interaction volume across avatar conditions (*N* = 42).

Measure	Cat-like	Human-like	Object-like	F (2, 82)	*p*
Free-conversation duration (min)	7.61 ± 0.96	7.50 ± 0.98	7.42 ± 0.99	1.52	0.22
Total word count	755 ± 102	740 ± 100	725 ± 101	2.10	0.13
Total dialogue turns	19.2 ± 2.4	18.9 ± 2.5	18.7 ± 2.5	1.20	0.31
User utterances	9.7 ± 1.1	9.5 ± 0.9	9.4 ± 1.6	1.05	0.35
System responses	9.5 ± 1.3	9.4 ± 1.1	9.3 ± 0.4	0.95	0.39

Notes. A user utterance was defined as a continuous segment of participant speech. Turn segmentation followed standard dialogue-partitioning conventions: a new utterance was recorded when (a) speech activity transitioned to the system or (b) a pause exceeding 2 s occurred within participant speech. A system response was defined as each complete output generated by the virtual doctor following participant input. Each uninterrupted system-generated text block was counted as one response. Total dialogue turns were calculated as the sum of user utterances and system responses within the free-conversation interval. All counts represent integer frequencies per participant per condition.

**Table A3:** Normalized Face AOI metrics.

Avatar	Width × Height	Area (px^2^)	% of Screen	Pro_Face_FD (M ± SD)	Pro_Face_FC (M ± SD)
Cat-like	370 × 270	99,900	1.13%	0.577 ± 0.050	0.571 ± 0.051
Human-like	400 × 440	176,000	1.99%	0.509 ± 0.053	0.480 ± 0.072
Object-like	740 × 600	444,000	5.02%	0.312 ± 0.065	0.337 ± 0.061

Notes. AOIs were defined using fixed rectangular bounding regions encompassing the eyes, mouth, and overall facial contour of each avatar. Screen resolution was 4096 × 2160 pixels (8,847,360 total pixels). “Pro_Face_FD” denotes the proportion of total fixation duration (Face + Text AOIs) allocated to the Face AOI, and “Pro_Face_FC” denotes the proportion of total fixation count allocated to the Face AOI.

## Appendix C. Structured Summary of the System-Level Prompt

The system prompt defined the behavioral and ethical constraints of the virtual psychologist and was applied identically across all avatar conditions.

1. Role Framing

The model was instructed to adopt the role of a professionally trained psychological counselor, primarily grounded in cognitive-behavioral therapy (CBT) and mindfulness-based approaches. The tone was specified as warm, accepting, and supportive while maintaining professional boundaries.

2. Interaction Principles

The prompt required that the model:

Reflect the user’s emotional content before guiding further exploration;

Avoid prescriptive or absolutist language (e.g., “you should”), instead framing suggestions tentatively (e.g., “you might consider…”);

Provide brief reflective summaries or small, actionable suggestions at the end of exchanges when appropriate.

3. Ethical Safeguards

The system was instructed to provide crisis-resource recommendations if users expressed explicit crisis-related content and to clarify that the system does not replace professional medical or psychological care.

4. Response Constraints

Responses were constrained to approximately 100 words, with consistent tone and structure across all conditions. Generation parameters (temperature, top_p, max_tokens, and penalties) were fixed throughout the experiment, as specified in the Methods section.
